# Aging conundrum: A perspective for ovarian aging

**DOI:** 10.3389/fendo.2022.952471

**Published:** 2022-08-19

**Authors:** Jiachen Wu, Yang Liu, Yinhua Song, Lingjuan Wang, Jihui Ai, Kezhen Li

**Affiliations:** Department of Obstetrics and Gynecology, Tongji Hospital, Tongji Medical College, Huazhong University of Science and Technology, Wuhan, China

**Keywords:** oocyte, ovary, aging, ovarian aging, interventions

## Abstract

Progressive loss of physiological integrity and accumulation of degenerative changes leading to functional impairment and increased susceptibility to diseases are the main features of aging. The ovary, the key organ that maintains female reproductive and endocrine function, enters aging earlier and faster than other organs and has attracted extensive attention from society. Ovarian aging is mainly characterized by the progressive decline in the number and quality of oocytes, the regulatory mechanisms of which have yet to be systematically elucidated. This review discusses the hallmarks of aging to further highlight the main characteristics of ovarian aging and attempt to explore its clinical symptoms and underlying mechanisms. Finally, the intervention strategies related to aging are elaborated, especially the potential role of stem cells and cryopreservation of embryos, oocytes, or ovarian tissue in the delay of ovarian aging.

## Introduction

Ovarian aging is characterized by the gradual decline in the quantity and quality of oocytes, mainly due to the low number of primordial follicles (PMFs) at birth and high monthly depletion during the reproductive period (1, 2). Ovarian aging manifests as reproductive decline until the loss of fertility, accompanied by endocrine dysfunction, menstrual cycle abnormalities, and other clinical symptoms (3, 4). Ovarian aging includes age-related physiological aging and pathological failure caused by different factors (5, 6). Age-related ovarian aging is a natural and inevitable physiological aging process. The age-dependent decline in oocyte quality accelerates between 35 and 40 years, and the natural menopause transition usually occurs between 40 and 45 years, with an average age of menopause between 50 and 52 years (7). In terms of pathological failure, ovarian aging mainly refers to premature ovarian insufficiency (POI), which is divided into primary or secondary POI (6, 8). POI affects approximately 1 percent of women under 40 years of age and 0.1 percent of women under 30 years of age (9). However, the etiology of primary POI remains unclear and may involve chromosomal abnormalities, gene mutations, enzyme deficiencies, and autoimmune disorders (10, 11). Secondary POI is associated with factors such as unhealthy lifestyles, chemotherapy, radiotherapy, reproductive system surgery, surgical menopause, endocrine disrupting chemicals, viral infection, or certain infectious diseases (12). This review focuses on the histomorphology and function of the ovary, clinical symptoms, pathogenesis and intervention strategies of ovarian aging, aiming to reveal the key regulatory factors of ovarian aging in the subfertility period.

## Hallmarks of aging

**Figure 1:**
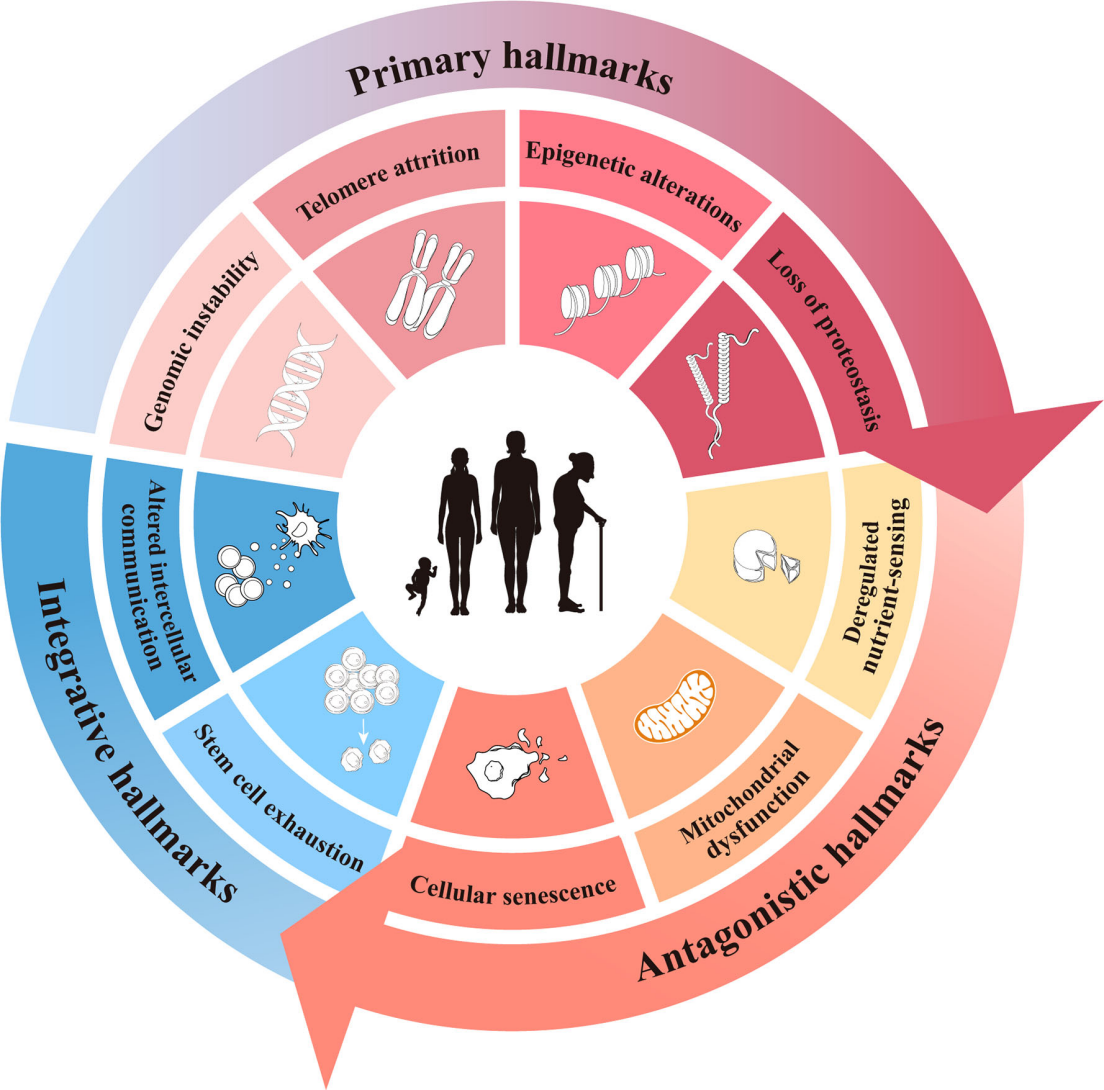
The current hallmarks of aging.

Aging can be defined as the progressive accumulation of degenerative changes that ultimately leads to an increased probability of functional impairment and mortality. Lopez-Otin C and Blasco MA et al. proposed that the current molecular and cellular hallmarks of aging be grouped into three categories: primary, antagonistic, and integrative hallmarks (13). While these hallmarks of aging have been presented as nine separate hallmarks in various research disciplines and there is a certain degree of cross-linking between them, we still hoped to summarize them by hierarchical relationship. Here, the hallmarks of aging are reviewed to provide some insight into the initiation of ovarian aging (**Figure 1**).

### Primary hallmarks

The primary hallmarks are considered to be the actual cause of aging and have a clear negative impact on deoxyribonucleic acid (DNA) (14). They first initiate cell damage, resulting in cumulative damage and gradual loss of function over time, which manifest as genomic instability, telomere attrition, epigenetic alterations, and loss of proteostasis (15–18).

Sustaining genome integrity requires the integration of multiple mechanisms and signaling pathways, and its stability is crucial for individual growth and human health (19). Genome instability is an increasing trend of genomic alteration during the cell life cycle, driven by a variety of endogenous and exogenous damage (20). Furthermore, genomic instability is reflected in gene mutation, replication, and transcription blockage, as well as DNA repair defects, which lead to the decline of organ function and disease progression, such as xeroderma pigmentosum, Cockayne syndrome, and other aging-related diseases (21–23).

The main function of telomeres is to protect the ends of chromosomes to maintain genomic integrity (24–26). Age and environmental factors such as inflammation, reactive oxygen species (ROS), and exposure to radiation or toxins can accelerate telomere attrition (27). The indispensable components in telomere maintenance, such as telomerase, telomerase ribonucleic acid (RNA) components, and shelterin complex, are closely correlated with aging and age-related diseases, such as premature aging syndromes and cancer (28).

Epigenetic alterations in DNA methylation, histone modification, and chromatin remodeling affect aging and longevity (29). Among various epigenetic alterations, DNA methylation is directly related to ROS metabolism through key redox intermediates such as 2-oxoglutarate, S-adenosylmethionine (SAM), and nicotine adenine dinucleotide (NAD) (30). These intermediate fluctuations directly affect epigenetic characteristics, resulting in detectable changes in gene expression and protein modification. Studies have shown that mouse quiescent satellite cells and teleost fish brain tissues display epigenetic repression of trimethylated histone H3 lysine 27 (H3K27me3) during aging (31, 32). Another study showed that the trimethylated histone H3 lysine 4 (H3K4me3) complex regulates the lifespan of Caenorhabditis elegans (33, 34). In addition, epigenetic alterations during aging also cause obvious chromatin structural changes, including heterochromatin region loss, global histone loss, and chromatin spatial interaction changes (35).

In addition, the maintenance of proteostasis is the key to ensuring normal organism development, resisting environmental stress, and promoting healthy aging and longevity (36, 37). As part of the proteostasis network, the ubiquitin-proteasome system and the autophagy-lysosome pathway are two major mechanisms of intracellular protein degradation (38–40). Studies have shown that the failure of autophagy in physiological aging satellite cells causes the loss of proteostasis, increased mitochondrial dysfunction, and ROS and ultimately leads to a decline in satellite cell number and function (41). In addition, loss of protein solubility and accumulation of aggregates are the histopathological hallmarks of several neurodegenerative diseases, such as Parkinson’s disease and Alzheimer’s disease (42–44).

### Antagonistic hallmarks

Antagonistic hallmarks, including cell senescence, mitochondrial dysfunction, and deregulated nutrient sensing, respond to the damage caused by primary hallmarks and are considered to be part of the compensatory or antagonistic response to damage. These responses initially mitigate the damage, and if enlarged or aggravated, they ultimately become deleterious and cause further damage.

Cell senescence is a generally irreversible and permanent state in the cell cycle caused by different stresses (45), including continuous DNA damage, irradiation, ROS, and viral infection (46). Cell senescence is accompanied by a low response to mitogenic stimuli, an inability to re-enter the cell cycle, and an enhanced secretory phenotype (47). The main structural and functional changes in aging cells include decreased membrane selective permeability, decreased responsiveness to mitogenic stimuli, disordered immune function, reduced enzyme activity, enhanced lysosomal activity, and decreased antioxidant capacity (48, 49). Cell senescence can damage tissue repair and regeneration, leading to age-dependent diseases, such as osteoporosis, pulmonary fibrosis, renal diseases, hepatic steatosis, and cardiovascular and neurodegenerative diseases (50).

As the primary site of oxidation of carbohydrates, fats, and amino acids to produce adenosine triphosphate (ATP), the quality and activity of mitochondria are essential for homeostasis maintenance, cell cycle control, and programmed cell death (51–53). Mitochondrial signaling pathways involving aging have been studied, such as mitochondrial dynamics, mitochondrial protein synthesis, mitochondrial autophagy, oxidative phosphorylation, ROS, and mitochondrial DNA damage (54, 55). Severe mitochondrial dysfunction can lead to biosynthesis disorders, insufficient energy supply, and increased ROS, thereby aggravating tissue and organ damage and even causing a variety of aging-related pathological changes (56–58).

The ability of cells to respond to nutrient-sensitive signaling pathways is tightly linked with nutrient availability and metabolic homeostasis, affecting the acquisition and maintenance of cell growth, cellular senescence, metabolism, and other physiological processes (59–62). Multiple signaling pathways are known to be involved in the regulation of nutrients, especially insulin/insulin-like growth factor 1 (IGF-1), the mammalian target of rapamycin (mTOR), and adenosine monophosphate-activated protein kinase (AMPK) signaling systems (63, 64). However, the dysregulation of nutrient sensing and energy metabolic pathways is closely related to metabolic diseases, such as obesity, type 2 diabetes mellitus, metabolic syndrome, and other age-associated diseases (65, 66).

### Integrative hallmarks

Stem cell exhaustion and altered intercellular communication are thought to be integrative hallmarks because they directly affect tissue homeostasis and function. When the cumulative damage caused by the primary and antagonistic hallmarks cannot be compensated for through tissue homeostatic mechanisms, integrative hallmarks arise and inevitably lead to the functional decline associated with aging.

Stem cell exhaustion, including the loss of stem cell number or function, is a progressive process and a comprehensive result of multiple aging-related injuries, leading to sustained and irreversible changes in the inherent features of stem cells (67–69). Throughout the life cycle, tissue-derived adult stem cells are essential for tissue homeostasis maintenance and regeneration by balancing self-renewal with lineage selection (70, 71). Therefore, adult stem cell exhaustion is considered to be an important driving factor behind the decline in tissue and organ function observed during aging. Beyond intracellular autonomous changes, aging also changes intercellular and intertissue communication (13, 72). These communications involve multiple independent or simultaneous processes that depend on physiological or pathological conditions to affect and maintain tissue homeostasis (35). Altered intercellular communication in the immune system is a progressive exacerbation of the proinflammatory state and reduced immune surveillance and immune response (73–75). As an acute and transient response to harmful conditions, the inflammatory response is conducive to the defense, repair, turnover, and adaptation of many tissues (76). However, with age, the innate and adaptive immune systems change (77). A chronic and low-grade inflammatory state termed inflammaging is likely to have a detrimental effect on the effectiveness of the immune system (78, 79). Studies have shown that changes in redox balance, increased senescence-associated secretory phenotype (SASP), and reduced effective autophagy trigger inflammasomes, suggesting that aging-related diseases and aging itself may be delayed by inhibiting proinflammatory molecular mechanisms (80).

## Histomorphological and functional changes in the ovary at different stages

### Histomorphological aspects of the ovary

In a study of human ovarian histomorphology, the ovaries of fetuses at 9-40 weeks of gestation are mostly almond-shaped and arranged obliquely in the fetal period (81). In the abdominal cavity, the ovaries usually descend slowly from the anterior ureter and above the common iliac artery with increasing volume until after birth. The ovaries have a smooth surface without ovulation until puberty, and menarche is an important sign of the onset of ovulation. In adolescence, the ovaries have a grayish-white, flattened oval appearance, begin to ovulate, and gradually have periodicity (82). Due to the extrusion of oocytes, the ovarian surface becomes uneven, and empty ovulation can be observed (83). In the absence of pregnancy, the ovaries of healthy adult women undergo extensive dynamic tissue remodeling during each menstrual cycle throughout the reproductive period (approximately 40 years) (84). The ovary consists of four layers from the outer to the central section: the germinal epithelium layer, the nonvascularized and thick fibrous-rich layer called tunica albuginea, the cortex containing ovarian follicles, and the medulla containing loose connective tissue and blood vessels (85). Studies have shown that ovarian fibrosis and stiffness increase with age in the mammalian ovary and depend on the age-related increase in collagen and the decrease in hyaluronan matrices (86, 87). As women enter the perimenopausal period from the reproductive period, obvious morphological and structural degeneration of the ovary occurs. Owing to the decrease in the number and diameter of follicles, aging ovaries shrink and show a wrinkled, nonglossy appearance (88).

Ovarian follicles are structural and functional units of the ovary, in which somatic cells and germ cells are well interrelated and interdependent (89). Human primordial germ cells differentiate into oogonia and proliferate, and this differentiation occurs continuously through mitosis and meiosis, stopping at the diplotene stage of meiotic prophase I (MI), and may last for decades until the oocyte is ovulated (90). Follicular development includes oocyte development, extensive proliferation and differentiation of granulosa cells (GCs), and theca cells with highly vascularized and specialized tissue layers generated by stromal cells (84). At the 20th week of fetal development, approximately 6-7 million oocytes in the ovary are surrounded by a layer of flat granulosa cells to form PMFs (91). After that, most PMFs are rapidly lost *via* apoptosis in the second half of fetal life, leaving only 1-2 million PMFs at birth (92). After birth, this high rate of follicle loss slows somewhat, and some PMFs can be recruited into the growing follicular pool and develop into antral follicles, most of which will inevitably enter the atresia stage. Most PMFs undergo degeneration or atresia at any stage of ovarian folliculogenesis. With the help of HPO axis regulation, ovarian function gradually matures, and approximately 300,000-400,000 PMFs are retained at menarche (93). Menstruation gradually became regular from the beginning of irregularity, with the decline of PMFs stabilized, but then gradually accelerated (94). Only 400-500 follicles reach the ovulatory phase during the reproductive span of healthy women (84). The ovulation process involves the expansion of the oocyte-cumulus complex, digestion of the follicle wall, resumption of oocyte meiosis, extrusion of meiotic prophase II oocytes, remodeling of the extracellular matrix, etc. (95–97). Among them, cumulus expansion and oocyte maturation are the key processes of ovulation (98).

### Functional aspects of the ovary

The ovary has both reproductive and endocrine functions. Ovarian reproductive function is mainly controlled by the hypothalamic-pituitary-ovarian (HPO) axis during the regular menstrual cycle (99). Ovarian endocrine function involves the secretion of steroid hormones, including estrogen, progesterone, a small amount of androgen, and various cytokines (100). These substances affect the development and function of the female vagina, uterus, oviduct, breast, and other organs.

Ovary reproductive function is the result of numerous interactions, among which follicular reserve plays a fundamental role. It is known that the PMF pool constitutes the ovarian reserve, and both reproductive and endocrine functions are limited by the PMF pool (4). Multiple cellular components of follicles coordinate and interact to regulate ovarian hormone secretion and affect oocyte development and maturation. As the number of follicles decreases, the quality of oocytes diminishes as well, especially after the age of 35 years (93). Research has shown that the decline in follicle numbers is a biexponential function of age, and this change occurs at the critical value of 25 000 follicles at the age of 37.5 years (94). The menopause transition marks a period of physiological change, during which the ovaries undergo incremental natural aging, and the PMF pool experiences a continuous irreversible decline (101). Subsequently, the rate of ovarian aging was unexpectedly accelerated. At the age of 51 years, the number of follicles decreased to 1000, which can be used as a threshold for menopause, as it corresponds to the average age of menopause in women (93, 102).

Ovarian endocrine function is jointly affected by ovarian sympathetic innervation, feedback regulation of the HPO axis, and complex interactions of the hormone axis (99). Follicles are not only the source of supply for female germ cells but also secrete essential hormones necessary to maintain normal endocrine function (103). The accelerated depletion of follicles in the PMF pool during the subfertility period may be related to increased sympathetic nerve excitement. Currently, studies have confirmed the presence of sympathetic innervation of the ovary, including the ovarian plexus nerve (projecting to the ovarian vasculature) and the superior ovarian nerve (projecting to the follicle), which can regulate ovarian blood flow and directly regulate steroid hormone production (104).

Ovarian aging is linked to changes in the HPO axis and a progressive decline in ovarian endocrine function, especially disorders of sex hormone levels. Among many sex hormones, anti-Müllerian hormone (AMH) is still the preferred ovarian reserve indicator in various clinical situations (105). AMH is produced by granulosa cells of small antral follicles in the ovary, not controlled by the hypothalamus or by gonadotropins, and independent of the menstrual cycle (106). A clinical trial showed that serum AMH levels in women aged 21-41 years declined by 5.6% per year (107). In addition, the complex interaction of follicle-stimulating hormone (FSH) and luteinizing hormone (LH) in the hypothalamus, anterior pituitary gland, and reproductive organs has complementary effects on ovarian folliculogenesis and ovulation (108). With age, women will experience early menopause transition, characterized by fluctuations in estrogen levels but generally sufficient, and ovulation generally occurs during the menstrual cycle (109). Finally, menopause is the final manifestation and hallmark event of ovarian aging, which manifests as a permanent cessation of the menstrual cycle and a serious decline in hormone secretion after the loss of follicular activity, especially the decline in estrogen levels (110).

## Clinical symptoms and mechanisms of ovarian aging

### Clinical symptoms of ovarian aging

The female reproductive system, especially the ovaries, is aging before any other organ system. This phenomenon has obvious clinical significance and may lead to infertility, abortion, birth defects, menstrual cycle disorders, or even amenorrhea and systemic deterioration caused by estrogen deficiency (111). A cohort study of 751 women who were artificially inseminated showed that the probability of pregnancy decreased rapidly after 31 years of age, and the probability of adverse pregnancy outcomes began to increase (112). In addition, ovarian aging may manifest as a shortened or prolonged menstrual cycle, irregular cycle, excessive or insufficient menstrual volume, and perimenopausal abnormal uterine bleeding (113). Ovarian aging leads to estrogen deficiency, which not only directly affects the tissues and organs with estrogen receptors, such as the ovary, endometrium, vaginal epithelium, skin, hypothalamus, and urinary tract but also influences other aspects of the organism, including the cardiovascular, musculoskeletal, and immune systems, emotional and sleep patterns, cognitive ability, and energy metabolism (114). For example, degenerative skin changes occur with estrogen deficiency, characterized by skin atrophy and accelerated skin aging, including collagen atrophy, elasticity and epidermal thickness decrease, elongation increase, wrinkles, and dryness (115). In the cardiovascular system, estrogen deficiency can downregulate the production of nitric oxide, reduce endothelial-dependent vascular function and adversely affect cytokine-mediated cell adhesion and antiatherosclerosis activity (116). Estrogen deficiency can also lead to bone loss, articular cartilage degeneration, and increased risk of fracture, possibly causing pain, loss of mobility, and the development of osteoporosis (117). In muscle, estrogen has a significant effect on the stability of muscle membranes and can reduce or delay leukocyte infiltration after muscle injury (118). In the central nervous system, decreased estrogen levels influence cognition, sleep and mood and affect many neuropsychiatric disorders, including Alzheimer’s disease, schizophrenia, and depression (119, 120).

### Potential mechanism of ovarian aging

The lifespan of the ovary depends on the delicate balance between the survival and death of oocytes. PMF activation is the basis for ovarian folliculogenesis and maintenance of fertility (121). However, damage during ovarian folliculogenesis, including the activation, recruitment, and development of early follicles, has a complex relationship with ovarian aging. In addition, depletion of the PMF pool caused by massive follicular atresia and periodic ovulation is the fundamental cause of ovarian aging.

Since the first use of [3H]-thymidine ([3H]-TdR) incubation to distinguish between slow-growing follicles and dormant PMFs, studies related to ovarian folliculogenesis have been conducted for more than 30 years. The regulatory mechanism of folliculogenesis, especially PMF activation, remains difficult to uncover due to the sophisticated process. Otsuka and Shimasaki demonstrated that the mitotic activities of oocyte-derived bone morphogenetic protein-15 and GC-derived kit ligand depend on the oocyte-GC communication system, which may play a pivotal role in ovarian folliculogenesis (122). Additionally, a series of protein and polypeptide hormones secreted by the pituitary gland, such as prolactin (PRL), growth hormone (GH), FSH, and LH, are essential for the activation and initial recruitment of PMFs and the development of growing follicles (123, 124). Studies also suggest that cytokines such as epidermal growth factor (EGF), transforming growth factor-alpha (TGF alpha), basic fibroblast growth factor (bFGF), and IGF-1 are involved in folliculogenesis and ovulation (125–128).

Forkhead box L2 (FOXL2), a winged helix/forkhead domain transcription factor, is preferentially expressed in the ovary, eyelids, and pituitary gland (129). Studies have shown that FOXL2 participates in multiple stages of ovarian development and function and the differentiation of pregranulosa cells (91). A sufficient number of pregranulosa cells expressing FOXL2 and primary oocytes arrested at the diploid stage of MI are two indispensable prerequisites for pregranulosa cells to break through germline cysts and move around primary oocytes (130). Additionally, research has demonstrated that the short-term treatment of mouse ovaries with a phosphatase and tensin homolog deleted on chromosome 10 (PTEN) inhibitor or a phosphatidylinositol-3-kinase (PI3K) activator could increase the phosphorylation of protein kinase B (AKT) and the nuclear export of downstream forkhead box O3 (FOXO3) protein, thereby effectively activating dormant PMFs (131, 132).

Obviously, abnormal follicular activation and atresia are the intrinsic mechanisms of ovarian aging, which may involve endocrine, paracrine, or autocrine signaling pathways. Follicular atresia is closely related to autophagy or apoptosis (133). Autophagy, a highly conserved intracellular process that maintains homeostasis by removing useless, senescent organelles and macromolecules, is a unique pathway to cell death as well as an adaptive response that promotes cell survival (134). Numerous autophagosomes and autophagic responses were reported to be mainly observed and detected in dead oocytes, especially in primordial and primary follicle oocytes (135). Cell apoptosis appeared only in GCs around the secondary or antral follicles, suggesting that both apoptosis and autophagy can mediate the onset of follicular atresia, while cell apoptosis may be the main form of postnatal follicular atresia (136).

Cell apoptosis, especially the intrinsic mitochondrial pathway, is regulated by B-cell lymphoma-2 (BCL-2) family proteins (137). Myeloid cell leukemia-1 (MCL-1), an antiapoptotic protein of BCL-2 family members, has a prosurvival effect in various cell types (138). MCL-1 deficiency activated apoptosis in early PMFs, increased markers of mitochondrial dysfunction and autophagy in growing oocytes, activated the apoptosis cascade reaction, and increased sensitivity to cell fragmentation in ovulated oocytes (139). Therefore, MCL-1 is considered to be a basic survival factor for maintaining the postnatal ovarian reserve, survival of growing follicles, and effective mitochondrial function of oocytes (139). Endogenous advanced glycation end products (AGEs) and the receptor for AGEs are expressed in luteinized and theca cells as well as GCs derived from ovaries, and AGEs can induce ROS chain reactions and increase inflammation, resulting in protein, lipid, and nucleotide damage during aging in the ovarian microenvironment (140–142). The accumulation of age-related AGEs in ovarian follicles triggers ovarian aging, which may be related to the regulation of AMH and AMHRII expression to affect ovarian reserve, reduce ovarian vascular supply and decrease glucose uptake in GCs (143, 144).

## Potential retrieval strategies for ovarian aging

Delaying childbearing among women has become a universal phenomenon due to sociodemographic, economic, medical, lifestyle and behavioral factors (145, 146). Human female fertility and reproductive lifespan decline with physiological aging and pathological failure, accompanied by a decline in birth rates and an increase in the number of childless adults (147–149). Attention to aging, especially ovarian aging, and finding potential retrieval strategies to improve fertility and healthy life expectancy has become an urgent task in the reproductive field (4, 150).

### Dietary interventions

Calorie restriction, the reduction of dietary intake to below energy requirements while maintaining optimal nutrition, is considered one of the most promising nutritional interventions to attenuate aging (151, 152). Despite its simplicity, a constant reduction in calorie or food intake is not easy to maintain in the long run. Recently, fasting-related interventions, such as prolonged fasting, time-restricted eating, and intermittent fasting, have emerged as alternatives to calorie restriction (153). Many studies based on animal models confirm that calorie restriction can delay the progression of diabetes, stroke, neurodegeneration, sarcopenia, and cardiovascular disease; reduce the cytotoxicity of chemotherapy; and alleviate immune system disorders (154, 155). In addition, calorie restriction can reduce the activation of PMFs and increase the number of quiescent PMFs in mice, which is beneficial to the protection of ovarian reserve and may be a potential way to delay menopause (156).

### GH/IGF-1 axis interventions

The mechanistic links between GH and aging mainly involve the evolutionarily conserved insulin/IGF-1 and mTOR signaling pathways that affect growth, immunity, metabolism, homeostasis and aging (157, 158). The secretion of both GH and IGF-1 peaks at puberty and gradually decreases in adulthood until only low levels are detectable in individuals aged ≥60 years (159, 160). IGF-1 levels *in vivo* are regulated by GH, and IGF-1 also has a negative feedback regulation on GH secretion (161, 162). Studies have shown that IGF-1 has important effects on the healthy growth and function of cells and tissue in model organisms (163–165). GH can not only directly affect human oocytes and cumulus cells but also indirectly influence oocyte quality and maintain oocyte DNA integrity by activating IGF-1 synthesis or promoting ovarian steroidogenesis (166–168). Studies confirm that the GH/IGF-1 axis not only inhibits ROS accumulation and apoptosis in GCs but also regulates steroidogenesis and follicular proliferation in polycystic ovary syndrome (169, 170).

### mTOR/S6 kinase pathway interventions

mTOR is a highly conserved serine/threonine-protein kinase (171). mTOR acts as a signal transduction center that integrates environmental and intracellular nutrients and growth factor signals and regulates various processes, including cell proliferation, metabolism, immunity, cellular senescence, and protein synthesis (172, 173). In addition, mTOR can phosphorylate and activate ribosomal protein S6 kinase (S6K), which is the key regulatory element of cellular transcript translation and protein synthesis (174). The AKT/mTOR signaling pathway controls ovarian folliculogenesis by maintaining the PMF pool, including PMF activation, GC proliferation, and oocyte-GC intercellular communication (175). Studies have shown significant activation of the AKT/mTOR/S6K signaling pathway in humans with POI (176). Therefore, regulating the AKT/mTOR/S6K signaling pathways, such as AKT activators and mTOR activators, can induce further follicular maturation and development in women with POI (176).

### AMPK or specific sirtuin interventions

AMPK, a serine/threonine-protein kinase, is one of the key energy sensors in eukaryotic cells and organisms and plays critical roles in regulating growth and reprogramming metabolic processes, such as autophagy, mitochondrial biogenesis, and lipid, cholesterol, and glucose metabolism (177). For example, AMPK activation in multiple tissues can autonomously and involuntarily induce autophagy, allowing cellular components to be recycled for energy production under nutrient-limited conditions (178, 179). With increasing age, the responsiveness of AMPK signaling decreases, and the aging process increases, leading to impaired maintenance of cellular homeostasis (180). Studies have confirmed that modulation of AMPK signaling can activate autophagy in GCs, affect human ovarian function, and lead to abnormal folliculogenesis (181). Furthermore, AMPK is necessary for the normal response to steroid hormones, and its intervention has potential significance for delaying ovarian aging (182).

Sirtuins are a family of NAD-dependent histone deacetylases that modulate cellular functions, such as genomic stability, mitochondrial biogenesis, cellular metabolism, autophagy or apoptosis, and the inflammatory response (183). Seven sirtuin isoforms catalyze specific lysine substrate deacetylation in mammals (184, 185). As a promising target for the prevention of aging-related diseases, sirtuin 1 is the most commonly studied isoform (186, 187). Several plant-derived polyphenol compounds, including resveratrol, butein, fisetin, and quercetin, can activate sirtuin 1 and exert beneficial effects on longevity (188). Studies have shown that resveratrol can enhance luteinization-related gene expression and ovarian progesterone secretion and improve the quality of cryopreserved ovarian tissue and embryo outcome in mice after transplantation through anti-inflammatory and antioxidant mechanisms (189, 190).

### Stem cell interventions

The exploration of stem cell and stem cell-derived extracellular vesicle therapy in reproductive medicine has shown great promise and availability in preclinical and clinical trials to delay, prevent or even reverse ovarian aging (191, 192). In preclinical trials, rhesus monkeys provide a suitable model for studying ovarian aging (193). Research has observed that using juvenile bone-marrow-derived mesenchymal stem cells (BM-MSCs) to treat macaques with ovarian aging can increase ovarian volume, strengthen hormonal regulation, and promote follicular regeneration in senescent macaques (194). Recently, several clinical trials using autologous BM-MSCs and allogeneic human umbilical cord-derived mesenchymal stem cells (UC-MSCs) in the treatment of patients with premature ovarian failure have demonstrated encouraging preliminary data in the rescue of overall ovarian function, as evidenced by increased ovarian volume, resumed menstruation, improved levels of estradiol and AMH, increased number of stimulating antral follicles, and alleviated menopausal symptoms (195–198).

### Embryos, oocytes, or ovarian tissue cryopreservation

The possibility of freezing oocytes and embryos has been available for a long time, and the first birth with thawed oocytes was achieved in 1983 (199). However, ovarian translocation and cryopreservation of embryos and oocytes are not suitable for prepubertal girls and women requiring urgent initiation of cancer treatment (85, 200). At present, ovarian tissue cryopreservation is a potential therapeutic option for ovarian function recovery in POI patients without ovarian stimulation or subsequent delay in the start of cancer treatment (201). The cryopreserved ovarian cortex can be thawed and autotransplanted, which has been proven to restore fertility, preserve ovarian endocrine function, and avoid the incidence of premature menopause, thereby delaying ovarian aging (202). However, the main challenges for ovarian transplantation are the massive loss of PMFs during ischemia and hypoxia and the risk of reintroduction of malignant cells with transplanted tissues (203, 204).

## Conclusion and perspectives

The ovary is the core reproductive organ of women and is crucial for maintaining normal reproductive and endocrine function stability. With the increase in lifespan expectancy, ovarian aging has gradually become a key health problem for women and is associated with a progressive age-related decline in the number and quality of oocytes. When these processes occur earlier or accelerate, their clinical correlation is the diminished ovarian reserve and/or triggers POI. Therefore, clarifying the hallmarks of aging, further studying the molecular mechanisms of ovarian aging and optimizing ovarian aging interventions are of profound significance for inhibiting aging-related diseases, reversing or preventing ovarian aging, and promoting female health and longevity.

## Author contributions

JW: Conceptualization, Writing-Original Draft; YL, YS: Resources, Data Curation; LW: Supervision, Project administration, Funding acquisition; JA, KL: Resources, Methodology. All authors contributed to the article and approved the submitted version.

## Funding

This work was supported by the Project of the National Natural Science Foundation of China (82101700).

## Conflict of interest

The authors declare that the research was conducted in the absence of any commercial or financial relationships that could be construed as a potential conflict of interest.

## Publisher’s note

All claims expressed in this article are solely those of the authors and do not necessarily represent those of their affiliated organizations, or those of the publisher, the editors and the reviewers. Any product that may be evaluated in this article, or claim that may be made by its manufacturer, is not guaranteed or endorsed by the publisher.
